# Hospital Patients' Sick Insurance

**Published:** 1920-07-24

**Authors:** G. G. Panter

**Affiliations:** Secretary of the Great Northern Central Hospital.


### HOSPITAL PATIENTS' SICK INSURANCE.

BY G. G. PANTER, SECRETARY OF THE GREAT NORTHERN CENTRAL HOSPITAL.

EVERY million of the population should be divided into four grades :

| | |
|---|---|
| 1. Those who should be accommodated in general wards ... ... ... ... ... ... ... ... | 500,000 |
| 2. Those who should be accommodated in cubicle wards ... ... ... ... ... ... ... ... | 200,000 |
| 3. Those who should be accommodated in private wards ... ... ... ... ... ... ... ... | 150,000 |
| Approximate total needing hospital treatment ... | 850,000 |
| 4. Persons not needing hospital treatment ... ... | 150,000 |
| | 1,000,000 |

It is computed that two hospital beds are needed for every 1,000 persons, therefore 1,700 would be provided for a unit of one million of the population. It is estimated that 10 per cent. of grade 1 need out-patient treatment, that is 50,000 individual cases annually, or about 300,000 attendances each year.

Each grade should be subdivided into adults and children under sixteen years of age, and the charge regulated (except in grades 2 and 3) according to the number in family.

| Grade | Families | Individuals | Remarks | Contributions payable per week | Annual Receipts |
|---|---|---|---|---|---|
| | | | | | £ |
| 1 | — | 99,000 | Single or married without family | 3*d.* each | 64,300 |
| | 30,000 | 90,000 | In families of 3 persons | 6*d.* per family | 39,000 |
| | 25,000 | 100,000 | ,, 4 ,, | 7*d.* ,, | 37,900 |
| | 20,000 | 100,000 | ,, 5 ,, | 8*d.* ,, | 34,000 |
| | 10,000 | 60,000 | ,, 6 ,, | 9*d.* ,, | 19,500 |
| | 5,000 | 35,000 | ,, 7 ,, | 9*d.* ,, | 9,750 |
| | 2,000 | 16,000 | ,, 8 or over | 10*d.* ,, | 4.300 |
| | | 500,000 | (includes 51 % adults) | | 209,350 |
| 2 | | 100,000 | Adults | 21*s.* each p.a. | 105,000 |
| | | 100,000 | Children under 16 | 10*s.* 6*d.* ,, | 52,500 |
| | | 200,000 | | | |
| 3 | | 75,000 | Adults | £2 2*s.* each p.a. | 157,500 |
| | | 75,000 | Children under 16 | £1 1*s.* ,, | 78.750 |
| | | 150,000 | Total from contributions ... ... | | 603,100 |
| | | | Less cost of collection 10 per cent. ... | | 60.310 |
| | | | Net income available from each million persons | | £542,799 |

Weekly or yearly contributions on the above basis by or on behalf of a person would make that person a subscribing member of the hospital organisation, and ensure admission to any hospital which may have been selected as the one to which admission might be desired in the event of hospital treatment becoming necessary. This selection would be made when a member joins, but arrangements could be provided for transfer at stated periods. When the need for treatment arises, a recommendation from the general practitioner concerned with the case would be required to accompany the application for admission, with a certificate that the case is one requiring active medical or surgical treatment in hospital. A certificate of membership of the hospital organisation would not be transferable from one person to another, and epidemics and notifiable infectious diseases would be excepted from the scope of the scheme.

Patients in grade 1 would, in addition to in-patient treatment, be eligible as out-patients, but grades 2 and 3 would not be admitted to this department.

Grade 3 patients could under the scheme have their own medical adviser to attend the hospital in consultation with the specialist if desired, and at other times.

Persons in grade 4 could have the benefits of the hospital facilities in special departments upon full payment and by special arrangement with their medical adviser.

The income maximum might be fixed for each grade as follows :

| | |
|---|---|
| Grade 1. Maximum adjusted income ... | £100 per annum |
| Grade 2. Maximum adjusted income ... | 200 per annum |
| Grade 3. Maximum adjusted income ... | 500 per annum |

The method of adjustment of income would be on a basis similar to the following (take a case of a patient applying from a household with three children with a total income of £500 per annum) :

| | | |
|---|---|---|
| Total income ... ... ... ... ... ... | | £500 |
| Less allowance for one parent or adult dependant ... ... ... ... ... ... | £80 | |
| Less allowance for first child ... ... ... ... | 50 | |
| Less allowance for second child ... ... ... | 30 | |
| Less allowance for third child ... ... ... | 30 | |
| | — | 190 |
| Adjusted income ... ... ... ... ... | | £310 |

The premiums could be collected by existing life and other insurance companies, who would, after deducting a commission, pay the amounts received over to a central board. These collecting arrangements would also apply to any hospitals and Charity Organisation Society offices, which should act as collecting agencies in addition to the insurance companies.

The use of insurance companies would save a great deal of expense (possibly the commission after the first year would be less than that allowed for), and the scheme would be likely to fructify more quickly than by new organisations.

The central board would pay capitation grants to the hospitals treating their members as follows :

| | |
|---|---|
| Grade 1. 1,000 beds @ £160 per occupied bed per annum ... ... ... ... ... ... ... | £160,000 |
| Grade 2. 400 beds @ £180 per occupied bed per annum, plus allowance of 33⅓ per cent. for medical fees ... ... ... ... ... ... ... ... | 96,000 |
| Grade 3. 300 beds @ £220 per occupied bed per annum, plus allowance of 100 per cent. for medical fees ... ... ... ... ... ... ... ... | 132,000 |
| 300,000 O.P. attendances at @ 2s. per attendance ... | 30,000 |
| Grant to general hospitals for dealing with accident or emergency cases other than members of the hospital organisation at the rate of £1,200 per annum per 100 beds occupied ... ... ... ... | 20,400 |
| Grant towards cost of building, renovations, and extensions at the rate of £4,000 per annum per 100 beds occupied ... ... ... ... ... ... | 63,000 |
| Grant towards Motor Ambulance Service at the rate of £120 per 100 beds occupied ... ... ... | 2,040 |
| | £508,040 |

| | Per annum £ |
|---|---|
| The foregoing table would show that the Central Board would receive ... ... ... ... | 542,790 |
| And of this amount would pay over to hospitals ... | 508,040 |
| Leaving a balance in their hands to meet cost of distribution and form Reserve Fund of ... ... | 34,750 |

Although only persons who voluntarily select to make provision for illness in time of health should be eligible for treatment in the voluntary hospitals, the income from the endowment funds of hospitals could be used in providing hospital policies for persons who wish to join the scheme but are unable to meet the whole cost,

**Hospital Patients' Sick Insurance**—(*continued*).

or to help policyholders while out of work or during illness. Persons who do not join the scheme would have to seek admission to State hospitals, except in cases of accident or emergency, for which provision is suggested. Also the gifts of subscribers and donors would be used to help necessitous cases.

It might also be found possible under this scheme to supplement the present work of hospitals by providing a home nursing service for medical and surgical cases for those patients in grades 1, 2, and 3, who supplement their contributions by an additional 10 per cent.

The benefits of the scheme should not become available to any member of the organisation until a sum equal to one year's premium had been paid by or on behalf of a patient, except cases treated by arrangement with local authorities, etc. Persons neglecting to join the organisation until overtaken by illness, and then joining, would pay the whole cost of treatment.

If such a body as King Edward's Hospital Fund or the British Red Cross Society would become the Central Board, funds might be available to form a basis for commencement, and to meet unforeseen contingencies.

This Board would have the right of inspection of hospitals, and on its General Committee would have one representative from each hospital with over 250 beds.

Special hospitals would be retained, and their beds used for cases referred from general hospitals at the request of the patient or by other arrangement, payment being made on the basis of the foregoing capitation rates.

Actuarial adjustment could be provided for the transfer from one grade to another of persons with varying incomes, or a refund of a percentage of payments in the cases of persons whose incomes had increased beyond the maximum of grade 3, and had thereby been removed from the scope of the scheme. This would only apply to persons who during their membership had received no hospital benefits.

The adoption of the scheme would make the hospital organisation available to all citizens needing its help, and thus remove the serious hardship which exists at present for the middle classes, this being done without injury to the medical profession.

It would also enable hospitals to increase their accommodation in accordance with the numbers of their patients, as one bed could be added for every additional 500 members who choose any particular hospital for subsequent treatment.

These notes leave many points untouched, but those dealt with might form a basis for the discussion of the general principle of voluntary insurance.

